# Progress in Neurology

**Published:** 1902-05-31

**Authors:** 


### Progress in Neurology.

(*Continued from page* 118.)

**Tumours of the Optic Thalamus.**—Michell Clarke[5] reports an additional case of a tumour involving the optic thalamus, and discusses their diagnosis. The characteristic signs are (1) a sensation of heat or burning in the opposite upper extremity, (2) a marked intentional tremor in the same limb, absent during rest, and exactly resembling, during movement, the tremor of disseminated sclerosis, (3) hemiplegia of the opposite side of gradual onset, affecting the arm only for a considerable time, and predominating in this limb throughout. Instead of an intentional tremor there may be movements of a choreiform type or like those of post-hemiplegic chorea. Intention tremor is also met with in tumours of the cerebellum, and of the corpora quadrigemina. In any case then in which this symptom is present the differential diagnosis has to be made between tumours situated in these three situations, but in most cases the other signs enable one to do so.

The explanation of this intention tremor is doubtful. Ferrier is of opinion that tumours in these three situations all implicate the superior cerebellar peduncle, and insomuch as experimental lesions of this tract in monkeys produce similar tremors, this symptom is similarly produced. On the other hand Sharkey says they are due to pressure on the pyramidal tract, and the symptoms certainly follow the sequence given by Sharkey, namely, tremors, paralysis, rigid contracture. But the balance of evidence is in favour of their being due to implication of the superior cerebellar peduncle, the course of events being that a tumour of the thalamus first affects the peduncle, and later in the course of growth presses on and more or less destroys the pyramidal tract, and thus the tremor appears first, and later disappears owing to the supervention of more complete paralysis and of spasmodic contracture.

**Tumour of the Superior Vermis of the Cerebellum.**—Gordinier [6] reports a case of a neuroglioma of this region. There was double optic neuritis passing on into atrophy, intense headache, vomiting, dizziness, slow cerebration, and gradual loss of memory. The focal symptoms were an ophthalmoplegia interna, with a double incomplete external ophthalmoplegia, nystagmus, a marked cerebellar gait, a coarse intentional tremor of the hands, and ataxia of the left leg. The intentional tremor and nystagmus resembled those of disseminated sclerosis, but scanning speech was absent, and the other symptoms negatived such a diagnosis. At the post-mortem examination a neuroglioma was found, taking its origin in the superior vermis of the cerebellum, which in its growth forward into the mid-brain had destroyed the superior medullary velum, the interior of the posterior quadrigeminal bodies, the superior cerebellar peduncles at their point of decussation, and the nuclei of the fourth, and partially that of the third, cranial nerves. Gordinier remarks that a distinction between corpora quadrigeminal and cerebellar tumours is at times absolutely impossible. In favour of the tumour being primarily located in the quadrigeminal region is the appearance of an external bilateral asymmetrical ophthalmoplegia, as the first symptom, the cerebellar ataxia occurring later. In favour of the lesion being primarily in the cerebellum and involving secondarily the corpora quadrigemina is the early appearance of a typical cerebellar gait, especially if combined with paralysis of the abducens or facial nerves of one or both signs, and followed by ophthalmoplegia of the above-described type. The intention tremor or choreiform movements are due to implication of the superior cerebellar peduncles.

**Tumours of the Corpus Callosum.**—Putnam and Wilson [7] record three cases of brain tumour involving the corpus callosum, and give a summary of our knowledge of the subject, based on a collection of the 38 hitherto reported cases. A great characteristic of tumours in this situation is the marked prevalence of mental change, and, in general, their early occurrence. This feature is as common as in the case of tumours of the frontal lobes. The mental changes occurred both when the tumour was situated in the anterior part and when it implicated the posterior portion of the corpus callosum. As a rule, impairment of memory, dulness, and intellectual failure were the marked feature, and in many cases changes are reported which strongly suggest general paralysis—as foolish or irrational behaviour, irritability, and indifference. Where alterations of the emotional tendencies were present these were far oftener of excitative than of depressive character, and outbreaks of violence and even maniacal excitement now and then occurred. General tumour symptoms —headache, nausea, and vomiting—occurred early in about half the cases, late in five, and were altogether absent in five cases. The knee jerk was occasionally lost. Convulsions without any general characteristics were present in about half the cases. Optic neuritis was absent in nine cases. Other cranial nerve complications are usually lacking, for obvious reasons. Paralysis of the limbs, of hemiplegic distribution was present in half the cases. Bilateral paralysis, which is a far more significant sign, was present in a quarter. In many of the cases, where no definite paralysis was present, there was weakness and helplessness, especially of the legs. The clinical course of these cases is important, mainly from its occasionally striking brevity. The corpus callosum, though a true commisural tract, is apparently not essential to the performance of all mental and bodily functions, for it has been found absent in persons who during life presented no abnormal nerve symptoms, and longitudinal section of it in dogs produces no paralysis or change in disposition or intelligence. It is therefore by no means clear how the above-described symptoms are brought about.

**Subcortical Tumour of Broca's Convolution.**—Stewart [8] has recorded a case of subcortical glioma of Broca's convolution which gave rise to dysarthria. The patient, a man of 37 years of age, between August and December, 1900, had 10 transient attacks of total loss of speech utterance. During the attacks he understood perfectly what was said to him, and knew what he wished to utter. During the last few attacks a slight "deadness" and numbness of the right index and middle fingers were momentarily felt. Optic neuritis and headache of a severe character now developed, and later there appeared facial spasm followed by weakness of the lower right facial muscles and right side of the tongue. Articulation now became difficult, first as regards right labials, later as regards all sounds (dysarthria), and finally a condition of complete inability to utter words (anarthria) resulted. On operation a subcortical tumour was found under the lower part of the left ascending frontal convolution and removed. The power of articulation began to return on the eighth day, and by the twenty-eighth was nearly normal. Handwriting which was at first incoördinate also became normal. The case well illustrates that a subcortical lesion of Broca's convolution gives rise to dysarthria and anarthria, and not to aphasia. There is no clinical difference between such a speech disturbance due to a subcortical lesion of Broca's convolution and that caused by a bulbar lesion, and a differential diagnosis is only possible by the help of collateral symptoms.

**Friedreich's Ataxy.**—Three cases have been recorded by Rankin,[9] who says that the diseases with which this complaint is most liable to be confounded are locomotor ataxy and disseminated sclerosis. It may be distinguished from the former by the age of the patient, the existence of the disease in other members of the family, the altered speech, characteristic foot deformity, tendency to spinal curvature, muscular tremors, and by the absence of visceral crises, Argyll Robertson pupils, lightning pains, and other sensory phenomena. Further, the gait in tabes is purely ataxic, whereas in Friedreich's ataxy it partakes of the cerebellar character as well and thus becomes a mixture of stamping and reeling. The resemblance to disseminated sclerosis is considerable. In both affections there are nystagmus, a difficulty of speech, muscular tremor, and an abnormal gait; but the nystagmus is earlier developed and more marked in disseminated sclerosis, and the gait is more spastic in disseminated sclerosis, which is not a family disease and rarely develops under 20 years of age. Again a marked feature of disseminated sclerosis is that the symptoms undergo periods of remission and exacerbation, unknown in Friedreich's ataxy. The etiology of Friedreich's ataxy is quite unknown; syphilis may be definitely excluded as a cause, though it may coincidentally exist. No more can be said in favour of consanguinity as a cause than that weakness of tissue inheritance is more likely to be intensified in the children of parents who come of the same stock than in those whose ancestry is derived from parents born of entirely different strains.

**Hereditary Cerebellar Ataxy.**—In 1893 Marie put forward the theory that cases hitherto described as Friedreich's ataxy could be divided into two groups, differing slightly from each other, and that one of the groups was dependent solely on cerebellar atrophy, whilst the other was caused by the already recognised spinal combined degeneration. According to Marie the features of Friedreich's ataxy proper are family type; inception before the sixteenth year; incoördination in station and progression rather of the staggering than the spinal ataxic sort, and often not increased by closure of the eyes; movement of the upper extremities resembling intention tremor, speech uncertain, slow, or explosive; loss of the tendon jerks; integrity of papillary reactions, ocular muscles and optic nerves; nystagmus; deformity of the feet and spinal curvature; unimportance of sensory and mental symptoms. Marie says that in hereditary cerebellar ataxy the type is hereditary as well as a family one; inception is after the age of 20; there is exaggeration, or at least presence, of the tendon jerks; defective papillary reactions; involvement of the optic nerves and ocular muscles, and absence of deformity. The other features are the same as in Friedreich's ataxy. Patrick[10] points out that the large amount of material, both clinical and pathological, which has been accumulated since 1893 does not bear out Marie's theory. In the first place no case has been recorded showing atrophy of the cerebellum solely. Secondly, the combined sclerosis of the cord in Friedreich's ataxy may or may not be associated with atrophy of the cerebellum, but the cases in which it is do not conform with Marie's cerebellar type.

**Family Periodic Paralysis.**—Buzzard[11] has recorded three most interesting cases of this rare disease, affecting a woman and her two sons. The mother, now about 35 years old, has suffered since childhood with attacks of paralysis, varying in severity from a comparatively unimportant disability lasting a few hours, to one in which for a day or two she is unable to move hand or foot. The attacks invariably commence during a period of rest, but generally follow a spell of physical exercise. During a severe attack all voluntary movements are quite impossible, other than those of the face, eyes, lips, palate, and those concerned in respiration, and often these are difficult. There is no pain during this condition of helplessness. The attacks at one time were as frequent as twice a week, but of late years they occur at increasingly longer intervals. The two boys, aged 13 and 11 years respectively, suffer in a similar way, the only difference being that the attacks are more frequent and of less severity, never lasting more than an hour or two; they have been noticed ever since they were first able to move about. They may occur as often as two or three times a day. The attacks almost invariably follow exercise, after an interval of rest. Examination of these patients revealed no signs of organic disease of the viscera or of the central or peripheral nervous systems. During an attack the superficial and deep reflexes were absent, and the muscles no longer responded to either faradic or galvanic stimuli of very considerable strength. The muscles affected are first and most often the proximal limb muscles, and the neck and trunk muscles also in severe attacks. The diaphragm always escapes even when the intercostal nerves are paralysed. It is only in severe attacks that the muscles supplied by cranial nerves are affected, and of these the levator palpebræ superioris is the earliest to show any sign. The simultaneous loss of irritability of the muscles during an attack to volitional, mechanical, galvanic and faradic stimuli strongly suggest that it is the muscle substance itself, irrespective of its neural connections, which is the seat of some temporary change. The cause of this change is unknown, but its almost invariable occurrence after exercise points to the presence of some poison thereby produced, or to want of removal by the lymphatics of some normal product of contraction, as the explanation. No means of alleviating or curing the disease has been discovered; but the attacks tend to occur less often as life advances.

[5] Brit. Med. Jour., Nov. 9, 1901. [6] Jour. of Nerv. and Mental Disease, Dec., 1901. [7] Jour. of Nerv. and Mental Disease, Oct., 1901. [8] American Medicine, Dec. 21, 1901. [9] Lancet, Jan. 18, 1902. [10] Jour. of Nerv. and Mental Disease, March 1902. [11] Lancet, Dec. 7, 1901.

